# Modern Imaging Techniques for Percutaneous Coronary Intervention Guidance: A Focus on Intravascular Ultrasound and Optical Coherence Tomography

**DOI:** 10.3390/jcm14248627

**Published:** 2025-12-05

**Authors:** Lorenzo Scalia, Mattia Squillace, Antonio Popolo Rubbio, Enrico Poletti, Federica Agnello, Antonio Sisinni, Francesco Bedogni, Marco Barbanti, Luca Testa

**Affiliations:** 1 Department of Cardiology, Umberto I Hospital, 94100 Enna, Italy; lorenzoscalia1993@gmail.com (L.S.);; 2 Department of Cardiology, IRCCS Policlinico San Donato, 20097 Milan, Italyluctes@gmail.com (L.T.); 3 HartCentrum, Ziekenhuis Netwerk Antwerpen (ZNA) Middelheim, 2020 Antwerp, Belgium; 4 Faculty of Medicine and Surgery, University of Enna “Kore”, 94100 Enna, Italy

**Keywords:** IVUS, OCT, percutaneous coronary intervention, intracoronary imaging

## Abstract

The use of imaging during percutaneous coronary intervention (PCI) can improve the outcomes by giving key information in every phase of the procedure. It can improve the knowledge of plaque composition thus helping the subsequent technical strategy; it can precisely define the measure of the stent to implant; it can assess in detail the correct positioning of the stent (apposition, expansion, and full coverage of the atherosclerotic plaque); it helps in recognizing the complications that may occur after stenting (e.g., edge dissection or tissue/thrombus protrusion in the stent area). Further, it could help evaluation for both diagnostic and therapeutic purposes of angiographic unknown or questionable findings [e.g., spontaneous coronary artery dissection (SCAD), characterization of mycotic aneurysm and pseudoaneurysm]. In the follow up phase, the use of intracoronary imaging may significantly improve the understanding of the mechanisms leading to the procedural failure. What this review adds is to describe the similarities and differences between intravascular ultrasound (IVUS) and optical coherence tomography (OCT) technologies, to highlight the evidence supporting their utility to improve PCI outcomes, to give practical advice and tools on daily interventional routine, to show a point of view on future perspectives and integration with artificial intelligence (AI).

## 1. Introduction

Nowadays, intravascular ultrasound (IVUS) and optical coherence tomography (OCT) represent the most used tools for coronary intravascular imaging.

These imaging techniques are considered such a helpful peri-procedural aid for both the strategy selection (e.g., plaque evaluation for lesion preparation) and the result optimization (e.g., tailoring eventual acute complications), thus permitting overcoming the limitations of angiography, optimizing procedural outcomes in a more standard way, and enhancing diagnostic accuracy.

**Figure 1:**
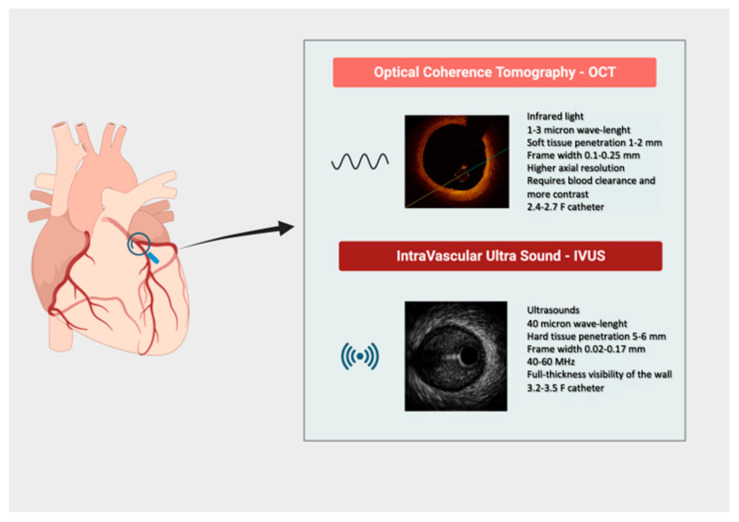
Synopsis of main technical characteristics of intravascular imaging tools.

Nevertheless, they are significantly different from a technical point of view due to distinctive physical characteristics: IVUS uses ultrasound (40 µm axial resolution at 40–60 MHz) with great tissue penetration and visualization of vessel structure, but with lower resolution when compared to OCT. Indeed, OCT uses infrared light (1–3-micron wavelength, corresponding to 1300 nm) with lower tissue penetration but higher resolution and consequent higher clarity [axial resolution (10–20 µm) and tissue penetration (~1–2 mm)] [1,2] (Table 1, Figure 1).

Evaluating the similarities and differences among these technologies is essential for guiding percutaneous coronary interventions (PCI). Moreover, both tools are still progressing due to improvements in catheter designs, advancements in imaging technologies, and evidence in the literature.

The aim of the present review is to summarize the current evidence regarding IVUS and OCT technologies in the setting of PCI, with the purpose of better understanding the selection process of these tools in clinical practice.

## 2. Methods

A comprehensive literature search was conducted in PubMed, Embase, and the Cochrane Library from January 2000 to September 2024. The following key terms and their combinations were used: intravascular ultrasound, optical coherence tomography, coronary stent, drug-eluting stent, percutaneous coronary intervention, and stented coronary lesions. Only studies published in English were considered. Eligible publications included randomized controlled trials, prospective or registry studies, and meta-analyses assessing IVUS- or OCT-guided PCI in stented coronary lesions. Studies focusing solely on non-stented or peripheral vascular interventions were excluded. In cases of overlapping study populations, the most comprehensive or recent dataset was retained. Reference lists of relevant articles and reviews were manually screened to identify additional eligible studies. All images and figures included in this review are either original or reproduced with permission from the respective copyright holders. No patient identifiers or personally recognizable information are included in any figures or datasets presented.

## 3. Clinical Studies Supporting Intracoronary Imaging

The influence of both intravascular imaging tools on clinical outcomes is well established in many studies and trials.

About that, prior studies showed how utilization of IVUS was associated with a lower risk of cardiovascular mortality and target vessel revascularization.

ADAPT-DES trial—The “Assessment of Dual Antiplatelet Therapy with Drug-Eluting Stents”, ADAPT-DES, was the first multicenter and prospective trial studying the clinical impact of IVUS after drug-eluting stent (DES) implantation that showed a reduction in 1-year rates of definite/probable stent thrombosis (ST) [0.6% vs. 1.0%; Hazard Ratio (HR) 0.40, 95% Confidence Interval (CI), 0.21–0.73; *p* = 0.003], myocardial infarction (MI) (2.5% vs. 3.7%; HR 0.66, 95% CI 0.49–0.88; *p* = 0.004), and major adverse cardiac events (MACE) (a composite of cardiac death, myocardial infarction, or stent thrombosis) (3.1% vs. 4.7%; HR, 0.70, 95% CI 0.55–0.88; *p* = 0.002) compared with angiography-guided DES implantation [3]. The benefit of IVUS-guided PCI on clinical endpoints was also maintained at 2-year follow-up [4,5].

ULTIMATE trial—In the three-year follow-up of the multicenter, randomized clinical trial ULTIMATE (Intravascular Ultrasound Guided Drug Eluting Stents Implantation in “All-Comers” Coronary Lesions), the investigators confirmed the lower rates of target vessel revascularization (TVR) (4.5% vs. 6.9%; *p* = 0.05) and ST (0.1% vs. 1.1%; *p* = 0.02), yet demonstrated at the one-year follow-up in a cohort of 1448 patients randomized to IVUS-guided PCI or angiography-guided PCI [6,7].

The IVUS-XPL—(Impact of Intravascular Ultrasound Guidance on the Outcomes of Xience Prime Stents in Long Lesions) was a randomized trial in which 1400 patients with long coronary lesions (stent length ≥ 28 mm) were assigned to either IVUS-guided or angiography-guided everolimus-eluting stent implantation [8]. The 5-year follow-up, completed in about 85% of participants (1183 patients), demonstrated that intravascular ultrasound guidance yielded a significantly lower rate of major adverse cardiac events (MACE) compared to angiographic guidance: 5.6% vs. 10.7%, corresponding to a hazard ratio of 0.50 (95% CI 0.34–0.75; *p* = 0.001) [9]. This benefit was largely driven by a reduction in ischemia-driven target lesion revascularization (4.8% vs. 8.4%; HR 0.54, *p* = 0.007). Importantly, a landmark analysis showed that the advantage of IVUS guidance persisted not only in the first year but also from years 1 to 5 (2.8% vs. 5.2%; HR 0.53, *p* = 0.031). These results indicate that the clinical benefit of IVUS-guided PCI is durable over long-term follow-up.

Medicare dataset cohort—A recent study aimed to explore the long-term outcome of IVUS-guided PCI in comparison to angiography-guided PCI. A total of 105,787 patients treated with an IVUS-guided PCI were extrapolated from a contemporary U.S. Medicare dataset cohort. In the propensity-matched analysis, the use of IVUS during PCI was associated with lower rates of long-term mortality, MI, and repeat revascularization [10].

Recently, the impact of IVUS on complex coronary lesions, defined as bifurcation, chronic total occlusion (CTO), left main (LM) disease, long-length lesion, multivessel PCI [11], multiple stent implantation, in-stent restenosis, or heavily calcified lesion, has been demonstrated. Among 1674 patients selected, after a median of 64 months of follow-up, IVUS-guided PCI was associated with a significantly lower risk of cardiac death [12].

RENOVATE-COMPLEX PCI trial—In the RENOVATE-COMPLEX PCI trial, a total of 1639 patients underwent randomization, with 1092 assigned to undergo intravascular imaging-guided PCI and 547 assigned to undergo angiography-guided PCI: the use of standard techniques for coronary artery-lesion preparation and stent implantation that were selected at the discretion of the operator [13]. Complex coronary artery lesions were defined as true bifurcation lesions, a CTO, unprotected LM coronary artery disease, long coronary artery lesions that would involve an expected stent length of at least 38 mm, multivessel PCI, and a severely calcified lesion. Among patients with complex coronary artery lesions, intravascular imaging-guided PCI led to a lower risk of a composite of death from cardiac causes, target vessel-related MI, or clinically driven target vessel revascularization than angiography-guided PCI: the primary end-point event had occurred in 76 patients (cumulative incidence, 7.7%) in the intravascular imaging group and in 60 patients (cumulative incidence, 12.3%) in the angiography group (HR 0.64; 95%, CI 0.45 to 0.89; *p* = 0.008).

Aiding precise assessment and guiding coronary interventions, the impact of OCT on clinical outcomes has been recognized in several studies, too.

CLI-OPCI study—The multi-center CLI-OPCI (Centro per la Lotta contro l’Infarto-optimization of Percutaneous Coronary Intervention) compared the clinical outcomes of conventional angiography-guided PCI with PCI guided by angiography plus OCT in 670 patients. At one-year follow-up, patients treated with OCT-guided PCI experienced significantly lower rates of cardiac death or MI and the composite of cardiac death, MI, and repeat revascularization at propensity-score adjusted analyses (9.6% vs. 14.8%, *p* = 0.044) [14].

DOCTORS trial—In the DOCTORS (Does optical coherence tomography optimize results of stenting”) randomized trial, the investigators compared OCT-guided PCI with angiography-guided PCI in 240 patients with non-ST-segment elevation acute coronary syndromes [15] (ACS), showing that the post-PCI fractional flow reserve (FFR) was higher in the OCT-guided PCI group when compared with the angiography-guided group (0.94 ± 0.04 vs. 0.92 ± 0.05, *p* = 0.005).

OPTICO-integration II trial—The introduction of real-time OCT with angiographic co-registration (ACR) allows a direct comparison of cross-sectional OCT images to angiography [16], and in addition, ACR may also be applied to IVUS. ACR-guided PCI was assessed in the “Impact of Real-time Angiographic Co-registered OCT on PCI Results” (OPTICO-integration II) trial that showed superiority of ACR OCT over angiography-guided and simple OCT-guided PCI in reducing the composite endpoint of major edge dissection and longitudinal geographic miss (GM) in the 84 prospectively randomized patients enrolled in this study [ACR-guided 4.2% as compared to OCT-guided PCI 19.1% *(p* = 0.03) and angiography-guided PCI 25.5% *(p* < 0.01)] [17]. Both the DOCTORS and OPTICO II trials primarily assessed physiological surrogates (such as FFR and geographic miss) rather than clinical outcomes, which represents a limitation of the analysis.

Intracoronary imaging modalities were also the object of a meta-analysis investigation.

Meta-analysis—As most of the study supports the role of IVUS and OCT in the post-stenting evaluation, a novel study evaluated pre-stenting IVUS assessment of the lesion to investigate the impact on clinical and procedural outcomes. According to this work in the literature, even though the pre-stenting IVUS group showed better acute procedural outcomes, this did not translate into a better clinical outcome at one-year follow-up [18] (Table 2).

In another updated meta-analysis embracing results from 22 trials, in which 15,964 patients were randomized and followed for a weighted mean duration of 24.7 months, the authors aimed to assess the comparative performance of intravascular imaging-guided PCI and angiography-guided PCI with DES [19]. Compared with angiography-guided PCI, intravascular imaging-guided PCI resulted in a decreased risk of target lesion failure (RR 0.71, 95% CI 0.63–0.80; *p* < 0.0001), driven by reductions in the risks of cardiac death (RR 0.55, 95% CI 0.41–0.75; *p* = 0.0001), target vessel—myocardial infarction (TV-MI) (RR 0.82, 95% CI 0.68–0.98; P = 0.030), and target lesion revascularization (RR 0.72, 95% CI 0.60–0.86; *p* = 0.0002). Intravascular imaging guidance was also found to reduce the risks of ST (RR 0.52, 95% CI 0.34–0.81; *p* = 0.0036), all MI (RR 0.83, 95% CI 0.71–0.99; *p* = 0.033), and all-cause death (RR 0.75, 95% CI 0.60–0.93; *p* = 0.0091). Outcomes were similar for OCT-guided and intravascular ultrasound-guided PCI.

To improve procedural outcomes, integrating anatomical and functional assessments may help to provide more accurate and tailored treatment in the setting of PCI; head-to-head comparisons between intravascular imaging and functional studies, such as FFR, are consequently a significant emerging area of research due to narrowed trial results. Following a detailed overview.

In the FORZA (Fractional Flow Reserve vs. Optical Coherence Tomography to Guide Revascularization of Intermediate Coronary Stenoses) trial, patients with angiographically intermediate coronary lesions (AICLs) were randomized 1:1 to either FFR guidance or OCT guidance for both PCI performance and, in the case of revascularization, PCI optimization [20]. In the FFR arm (*n* = 176), PCI was performed if FFR was ≤0.80, while in the OCT imaging arm (*n* = 174), PCI was performed if area stenosis was ≥75% or 50% to 75% with minimal luminal area <2.5 mm^2^ or plaque rupture. The primary endpoint of major adverse cardiac events or significant angina at 13 months occurred in 14.8% of patients in the FFR arm and in 8.0% in the OCT imaging arm *(p* = 0.048); the rate of medically managed patients was significantly higher *(p* < 0.001) and total cost significantly lower *(p* < 0.001) with FFR in comparison with OCT imaging. Thus, the study was underpowered for hard clinical endpoints, which limits the robustness of conclusions regarding major adverse events. Furthermore, the cost-effectiveness findings should be interpreted with caution, as they were derived from a single-center Italian cohort and may not be generalizable.

On 5-year follow-up analysis, compared with the FFR group, the OCT group had numerically lower rates of all-cause death (8.6% vs. 10.8%; HR 0.80, 95% CI 0.41–1.58; *p* = 0.525), MI (1.1% vs. 2.8%; HR 0.40, 95% CI 0.08–2.07; *p* = 0.275), and TVR (8.0% vs. 8.5%; HR 0.93, 95% CI 0.45–1.93; *p* = 0.854) [21].

As expected, the use of FFR reduced the number of PCIs as compared with OCT guidance. Periprocedural complications (e.g., kidney injury and periprocedural cardiac injury) were lower with FFR. Accordingly, an FFR-guided treatment strategy was associated with markedly lower resource utilization and lower costs than an OCT-guided strategy.

The PECTUS-obs trial (Identification of Risk Factors for Acute Coronary Events by OCT After STEMI and NSTEMI in Patients with Residual Non-flow Limiting Lesions) aimed to assess the association between OCT-identified high-risk plaques of FFR-negative non-culprit lesions and the occurrence of MACE [22]; in patients presenting MI, OCT was performed on all FFR-negative (FFR > 0.80) non-culprit lesions. A high-risk plaque was defined as containing at least 2 of the following prespecified criteria: (1) a lipid arc at least 90°, (2) a fibrous cap thickness less than 65 μm, and (3) either plaque rupture or thrombus presence. High-risk plaque is associated with a worse clinical outcome, which is mainly driven by a higher number of unplanned revascularizations (9.8% vs. 4.3%; *p* = 0.02), with a higher percentage of MACE at 2-year follow-up (HR 1.93, 95% CI 1.08–3.47; *p* = 0.02).

The PREVENT trial showed that preventive PCI of focal non-FFR-positive plaques that are angiographically >50% and have evidence of vulnerability on intravascular imaging, along with optimal medical therapy (OMT), is superior to OMT for clinical outcomes at 2 years. The trial sought to assess whether preventive PCI of non-flow-limiting vulnerable plaques (*n* = 803) improves clinical outcomes (a composite of death from cardiac causes, TV-MI, and ischemia-driven TVR) compared with OMT alone (*n* = 803) [23].

The trial was the first RCT to highlight the strategy of preventive PCI to treat vulnerable plaques, even if non-flow-limiting lesions, a paradigm-shifting but controversial concept. In patients with non-flow-limiting vulnerable coronary plaques, preventive PCI reduced major adverse cardiac events arising from high-risk vulnerable plaques compared with optimal medical therapy alone (absolute difference—3.0 percentage points (95% CI 4.4–1.8; *p* = 0.0003)), underlying the leading role of intravascular imaging on PCI guidance.

## 4. Characterization of Plaque Morphology

**Figure 2:**
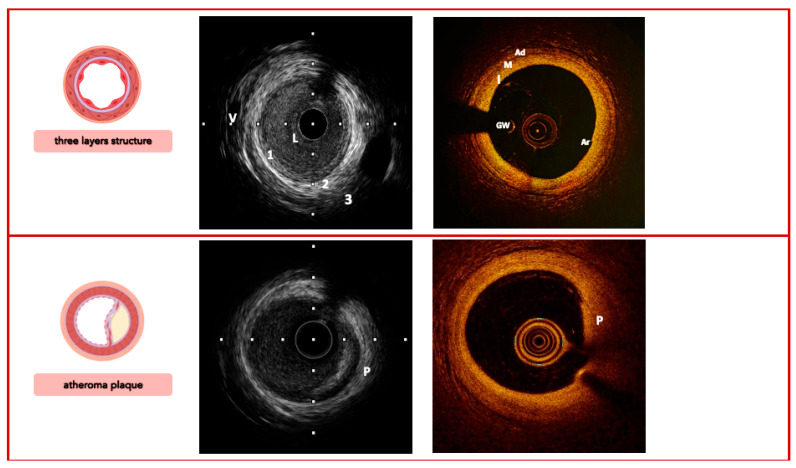
Imaging signatures inform on the coronary arteries’ three-layer structure (**top**) and characterize the morphology of a simplex atheroma plaque (**bottom**), comparing IVUS vs. OCT. *Legend:* 1/I, tunica intima; 2/M, tunica media; 3/Ad, tunica adventitia; V, vasa vasorum; L, lumen; GW, guidewire; Ar, artifact; P, plaque. Pullback speed of automated 1 mm/s for IVUS and 20 mm/s for OCT. IVUS frame rate of 30 frames/s and 150 frames/s for OCT.

**Figure 3:**
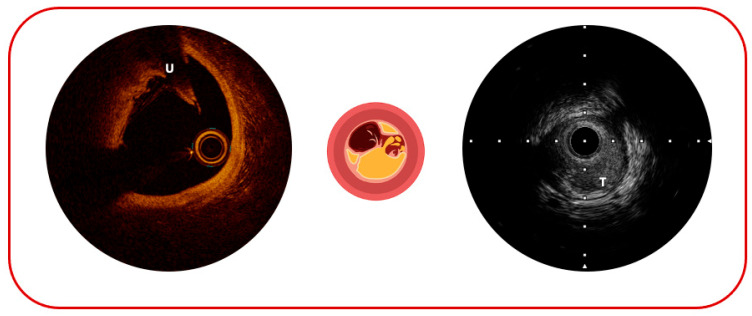
Possible complications of atherosclerotic plaque sequelae: on the (**left**), plaque ulceration (U); on the (**right**), plaque thrombosis (T).

**Figure 4:**
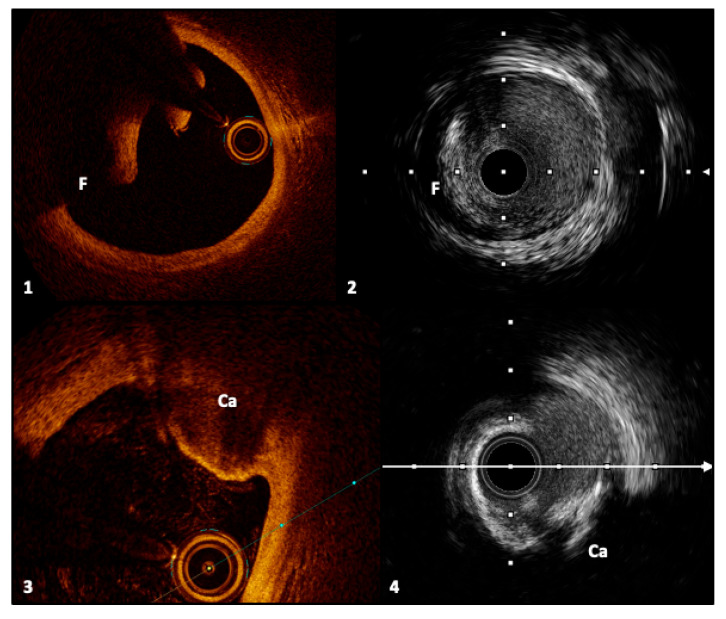
Frames 1 and 2, respectively, with OCT and IVUS, represent an intimal dissection flap with a false lumen. Frames 3 and 4 show a calcified nodule, described, respectively, with OCT and IVUS, characterized by signal-poor regions and acoustic shadowing. *Legend:* F, flap; Ca, calcified nodule. Pullback speed of automated 1 mm/s for IVUS and 20 mm/s for OCT. IVUS frame rate of 30 frames/s and 150 frames/s for OCT.

The anatomical trilaminar appearance of the vessel represents the starting point for imaging analysis of the plaque; the loss of normal anatomy is related to different types of atherosclerotic lesions with several histological modifications (Figure 2, Figure 3 and Figure 4).

The capacity to predict a plaque complication is one of the most argued conundrums and a fascinating topic in the field of interventional cardiology [24].

The concept of “plaque healing” and its implication in ACS have stimulated the development of many imaging studies aimed at understanding the pathobiology of atherosclerotic plaque and its dynamism [25].

### 4.1. Vulnerability of Coronary Plaque

Non-invasive intravascular imaging tools offer the potential to identify plaques with features of vulnerability, indicating patients at increased cardiovascular risk.

As a matter of fact, an in vivo OCT study demonstrated the presence of a similar plaque phenotype, higher prevalence of thin cap fibroatheroma, and lower prevalence of healed coronary plaques in patients with ACS compared with patients with stable coronary syndrome, whose plaques are characterized by a multilayered “onion-like” OCT appearance, considered a healing sign [26].

With IVUS, a large plaque burden, small lumen area, and positive remodeling have been demonstrated as strong markers of plaque ‘vulnerability’. Attenuated plaque or a necrotic core (especially with a non-detectable fibrous cap, <150 μm thick according to the resolution of the technique) by IVUS-virtual morphology is also a sign of plaque instability [27].

OCT, which has a ∼10-fold higher resolution, can categorize atherosclerotic lesions in the vessel wall and inside the lumen with high accuracy due to its great image resolution: low-attenuating, signal-rich lesions represent fibrous plaques; high-attenuating, signal-poor regions covered with fibrous cap represent lipid-rich plaques; and low-attenuating, signal-poor regions represent calcific plaques. Inside the lumen, the low-attenuating images represent white thrombus, and the high-attenuating images represent red thrombus.

OCT is considered superior for the detection of potentially vulnerable thin caps, remaining the most reliable imaging modality to confirm plaque erosion [28,29].

### 4.2. Plaque Morphology and Cardiovascular Events

The CLIMA study was the first study that has explored by OCT the correlation between the plaque morphology and the risk of future cardiovascular events: it was designed to explore the predictive value of multiple high-risk plaque features vulnerability in the same coronary lesion [minimum lumen area (MLA), fibrous cap thickness (FCT), lipid arc circumferential extension, and presence of defined macrophages]. Results showed how the combination of MLA <3.5 mm^2^, FCT <75 µmicron, lipid arc extension >180°, and the presence of macrophage infiltration conferred a high specificity with a negative predictive value of 96.9% for clinical endpoints such as MI [30].

Interestingly, Cao et al., in a recent work, adopted these high-risk criteria proposed in the CLIMA study to define “pancoronary vulnerability” in patients presented with ST elevation myocardial infarction and OCT diagnosis of plaque erosion or plaque rupture. Patients with culprit plaque erosion seem to have a limited “pancoronary vulnerability” compared to patients with culprit plaque rupture and high-risk criteria [31].

The application of near-infrared spectroscopy (NIRS) technology to IVUS (NIRS-IVUS) showed promising results concerning the recognition of plaque complications.

A maximum lipid core burden index of 4 mm (maxLCBI4 mm), plaque cavity, and convex calcium were detected by NIRS-IVUS analysis in patients with MI and compared with OCT analysis. High-risk plaque characteristics are specified as lipid-rich plaque, maximum plaque burden > 70%, and MLA < 4 mm^2^.

The PROSPECT II trial showed how combined NIRS and intravascular ultrasound detect angiographically non-obstructive lesions with a high lipid content and large plaque burden that are at increased risk for future adverse cardiac outcomes. The primary endpoint of cardiac death, MI, or unstable angina/progressive angina requiring revascularization and/or confirmed lesion progression occurred in 13.2% of all patients at 4 years. Most events occurred in the untreated non-flow-limiting lesions (8.0%), while culprit-lesion major adverse cardiac events occurred in 4.2% of patients [32]. The analysis showed how highly lipidic lesions were considered an independent predictor of patient-level non-culprit lesion-related MACEs (adjusted odds ratio 2.27, 95% CI 1.25–4.13) and non-culprit lesion-specific MACEs (7.83, 95% CI 4.12–14.89): the maxLCBI4 mm plays a prognostic role in detecting more vulnerable plaques, even though it actually does not have an interventional validation.

### 4.3. Calcified Lesions

NIRS-IVUS has demonstrated good specificity and sensitivity in identifying plaque erosion, rupture, and calcified nodules in acute myocardial infarction [33]. Among these, calcified nodules may increase the likelihood of plaque complexity and complications [34,35].

The IVUS detection of calcified nodules has been described as irregular calcification protruding into the vessel lumen. Their individuation by IVUS is considered an independent risk factor for cardiovascular events [36]. A post hoc analysis of the CLIMA registry established the relationship between their presence inside the plaque and the incidence of cardiac death and target lesion MI [37].

More broadly, the presence of diffuse calcification inside the intima and the media of the vessel represents a risk factor for low procedural success rates, higher risk of complications, and worse clinical outcomes [38,39,40].

In the MACE trial, a prospective, multicenter, observational clinical trial that enrolled 350 patients, in which patients were classified based on the severity of coronary calcified lesions, those with severe calcification had significantly worse outcomes at one-year follow-up [41].

Intracoronary imaging plays a pivotal role in the evaluation of calcification: IVUS can delineate the calcification arc and its thickness, whereas OCT is efficient and highly accurate to analyze both the calcification arc, thickness, and calcified plaque modification [2].

Nevertheless, IVUS is more accurate than OCT for the detection of coronary calcification, as demonstrated in a study by Wang et al., in which calcium was either not visible by OCT or was underestimated due to plaque attenuation [42].

Scoring systems, based on imaging techniques, have been validated to face calcified coronary lesions to define a better strategy for plaque modification prior to stent implantation.

In a retrospective study using 128 patients with pre- and post-stent OCT (then validated in an external cohort of 133 patients), a multivariable model showed that the independent predictors of stent expansion were a maximum calcium angle of 180°, maximum calcium thickness of 0.5 mm, and calcium length of 5 mm.

Based on these results, a calcium score (namely, the Fujino Score) was then defined as 2 points for maximum angle >180°, 1 point for maximum thickness >0.5 mm, and 1 point for length >5 mm, where the lesions with a score of 4 are at poor stent expansion risk [43].

In an analysis of 370 patients in which 31 calcified stenoses were selected after a standardized intravascular imaging algorithm with both IVUS and OCT, imaging-guided intravascular lithotripsy (IVL), based mainly on the Fujino OCT score, facilitated stent expansion and patient outcome [44].

More recently, Lampson et al. proposed the A.M.A.S.A. algorithm (Assessing the lesion crossability, Modify the calcium arc, Assess again, Stenting, Assess again after stenting) to manage calcified coronary lesions to define the best lesion preparation to gain procedural success [45,46].

Current calcium modification techniques—including rotational atherectomy (RA), orbital atherectomy (OA), and IVL—are essential for preparing heavily calcified coronary lesions prior to stent implantation. Intravascular imaging, particularly IVUS, plays a pivotal role in guiding these interventions by accurately delineating the depth, circumferential extent, and thickness of calcific deposits. These imaging insights inform both device selection and procedural parameters such as burr or crown size, rotational speed, and the need for adjunctive therapies. IVUS helps differentiate whether calcium is superficial, favoring ablation techniques like RA or OA to modify the intimal surface, or deep, where IVL may be required to induce fractures through the medial layer. Furthermore, imaging facilitates the strategic combination of complementary modalities to optimize plaque modification, enhance stent expansion, and improve procedural outcomes.

## 5. Procedural Guidance and Stent Sizing with Intracoronary Imaging

**Figure 5:**
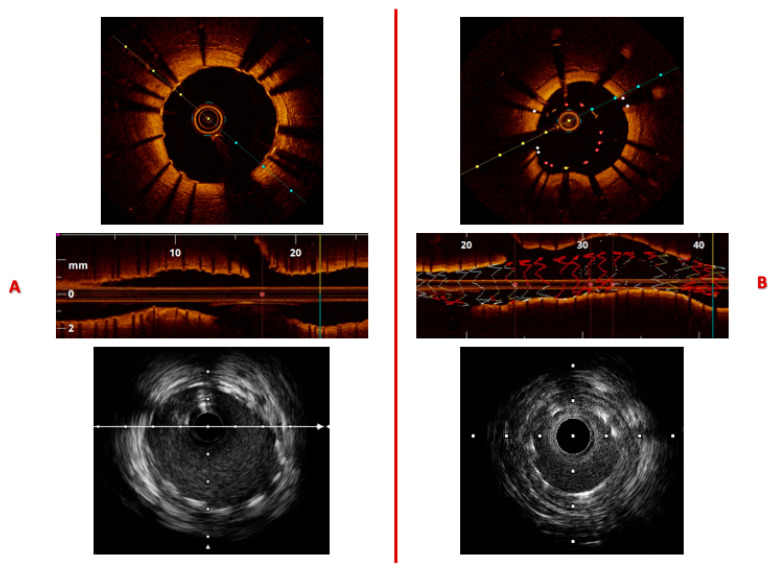
Layout (**A**) illustrates proper stent apposition and full expansion, as shown in the transverse and longitudinal OCT views, with a corresponding IVUS image displayed below. Layout (**B**) demonstrates stent under-expansion due to vessel caliber mismatch; a region of stent malapposition can also be observed between 20 and 40 mm along the upper vessel wall. Automated pullback speeds were 1 mm/s for IVUS and 20 mm/s for OCT, with frame rates of 30 frames/s for IVUS and 150 frames/s for OCT.

Intracoronary imaging can give precious information to select the appropriate and suitable stent size; imaging could also minimize some of the PCI complications, such as GM, thin cap fibroatheroma (TCFA) presence at reference segments, dissections, and the resulting risk of early ST and post-PCI target lesion failure (Figure 5).

It is consolidated that the stent sizing based on external elastic lamina (EEL) is preferable to a lumen-guided sizing because its result is a larger lumen area.

However, be careful, though intravascular imaging could be subject to several artifacts that can impact the accuracy of vessel measurements. Ring-down artifact, caused by reverberations within the transducer, appears as a bright halo near the catheter and can obscure the near-field, making it difficult to delineate the true lumen border. Guidewire shadowing occurs when the metallic guidewire blocks ultrasound waves, creating a dark acoustic shadow that hides part of the vessel wall and may lead to underestimation of lumen area or EEL dimensions. Additionally, near-field clutter from tissue motion or transducer interference can blur the proximal vessel wall, further complicating border detection.

The ILUMIEN trial was the first trial that demonstrated the non-inferiority of OCT-guided PCI to IVUS-guided PCI. In this trial, stent sizing was based on EEL measurement, particularly the smaller EEL diameter of the proximal or distal reference point. Using this strategy, OCT-guided PCI resulted in a similar minimum stent area to that of IVUS-guided PCI (the final median minimum stent area was 5.79 mm^2^ with OCT, 5.89 mm^2^ with IVUS, and 5.49 mm^2^ with angiography guidance) [47].

In the following ILUMIEN IV trial, researchers randomized 2487 patients at 80 sites in 18 countries to either OCT-guided PCI (*n* = 1233) or angiography-guided PCI (*n* = 1254). All patients had medication-treated diabetes and/or complex lesions. Overall findings showed that OCT-guided PCI led to a larger minimum stent area, enhanced the safety of the PCI procedure, and resulted in a nearly two-thirds reduction in ST over the two-year follow-up period [48]. However, OCT guidance did not reduce the two-year rate of target vessel failure, noted as the clinical main endpoint, compared with angiography-guided PCI because of a low and nearly identical rate of target vessel revascularization in the OCT-guided and angiography-guided PCI arms (Kaplan–Meier estimates, 7.4% and 8.2%, respectively; HR 0.90, 95% CI 0.67–1.19; *p* = 0.45). These factors suggest insufficient statistical power to detect group differences—indicating that longer follow-up or a larger sample size was needed for conclusive results—and may also reflect lower lesion complexity compared to previous trials.

The IVUS superiority to angiography for the PCI guidance in complex lesions has been established in the AVIO trial. In patients randomized to the IVUS-guided PCI group, stent expansion was evaluated. It was based on the optimal balloon size that should be used for post-dilatation, which was determined by averaging the media-to-media diameters of the distal and proximal stent segments and by the sites of maximal narrowing within the stent. If the stent implanted was considered underexpanded, post-dilatation was performed with a noncompliant balloon. With these IVUS criteria for stent optimization, a larger post-procedural minimal lumen diameter (MLD) was obtained [49].

The OCTOBER trial found routine use of OCT-guided PCI reduced adverse cardiac events with a lower incidence of MACE at 2 years in technically challenging patients with complex bifurcation lesions, compared with angiography-guided PCI and optional use of IVUS in LM bifurcations [50].

The OCTIVUS trial randomized 2008 patients with diverse coronary artery lesions to either OCT-guided PCI (*n* = 1005) or IVUS-guided PCI (*n* = 1003). In patients with significant coronary artery lesions, the trial showed how OCT-guided PCI was noninferior to IVUS-guided PCI with respect to the incidence of a composite of death from cardiac causes, target vessel-related MI, or ischemia-driven TVR at 1 year [51].

The OCCUPI trial expands the existing evidence base by examining outcomes in patients with both complex coronary lesions and challenging clinical scenarios. The primary endpoint—comprising cardiac death, ischemia-driven target lesion revascularization, myocardial infarction, and stent thrombosis—at 12 months—occurred in 5% of patients undergoing OCT-guided PCI compared with 7% in the angiography-guided group (HR 0.62; 95% CI 0.41–0.94; *p* = 0.023) [52]. These findings demonstrate that OCT-guided PCI provides superior cardiovascular outcomes at one year compared with angiography guidance, primarily driven by a significant reduction in ischemia-driven target lesion revascularization. However, this benefit was associated with increased contrast use and longer procedural times.

### Left Main Imaging Evaluation

Intrinsic anatomical features of LM—that confer to this coronary tract higher elastic properties, higher calcium content, and higher tapering—make the use of intravascular imaging an appealing solution to evaluate the plaque composition and to define the correct strategy to perform the most precise PCI [53,54].

The consensus statement of the European Bifurcation Club recommends IVUS guidance for LM elective PCI [55]. Meta-analysis of 10 studies showed that IVUS-guided PCI of LM reduced the risk of all-cause mortality by 40% and cardiac death by 53% [56]. Registry data have reported a reduced incidence of mortality, ST, and in-stent restenosis (ISR) over a period of five years with IVUS-guided LM PCI [57].

Recently, a clinical benefit has been demonstrated using an IVUS systematic protocol with well-defined targets for the LM PCI [58].

In a sub-analysis of the NOBLE trial (Nordic–Baltic–British left main revascularization study), LM target lesion revascularization resulted in being inferior in the group treated by IVUS-guided PCI (5.1% vs. 11.6%); nevertheless, this did not influence clinical outcomes [59].

The EXCEL IVUS sub-study (Evaluation of XIENCE versus Coronary Artery Bypass Surgery for Effectiveness of Left Main Revascularization) showed that patients who underwent IVUS-guided stent optimization experienced fewer cases of stent deformation and, consequently, lower rates of major adverse cardiovascular events (28% vs. 13.4%), LM-related myocardial infarction (18.9% vs. 4.6%), and LM ischemia-driven target lesion revascularization (19.6% vs. 7.7%) [60].

The LEft Main Oct-guided iNterventions trial (LEMON) was the first multicenter experience to report the feasibility and performance of OCT-guided LM PCI in 70 patients. Residual angiographic stenosis <50%, TIMI 3 flow in all branches, and adequate OCT stent expansion were the primary endpoints that were achieved in 86% of the patients, stating the feasibility and safety of OCT in this setting [61].

Advancements in technologies and evaluations in larger randomized trials may confer more solidity to OCT application in this field in the near future.

## 6. Intravascular Imaging Guidance of Coronary Stent Implantation

The strongest parameter to predict both ISR and ST is the post-PCI minimum-stent area (MSA) [62,63,64,65].

Several studies have demonstrated the strong link between ISR and stent under-expansion or malapposition related to the main procedure. Overall, most studies have defined several important factors (e.g., stent expansion, stent apposition, tissue protrusion (TP), edge dissection, and lesion coverage) for stent optimization when using intracoronary imaging tools [66,67,68].

### 6.1. Stent Expansion

On average, a greater absolute stent expansion is associated with better stent-related clinical outcomes and a lower risk of stent failure.

The cut-offs of IVUS MSA to optimize sensitivity and specificity of angiographic binary restenosis are similar for different types of DES: sirolimus-eluting stents 5.5 mm^2^, paclitaxel-eluting stents (PES) 5.7 mm^2^, everolimus-eluting stents 5.4 mm^2^, and zotarolimus-eluting stents 5.3 mm^2^ [69].

With OCT, the optimal cut-off to predict a post-procedural FFR of >0.90 was consistently found to be >5.44 mm^2^ in the DOCTORS trial; thus, the results from the Centro per la Lotta Contro L’Infarto-Optimization of Percutaneous Coronary Intervention (CLI-OPCI) II registries have revealed that an MLA of 4.5 mm^2^ is the best cut-off value for OCT to identify patients with adverse events [70].

### 6.2. Stent Malapposition

It refers to a contact gap of 200 μm or more between the stent struts and the vessel wall, as observed through OCT. This phenomenon could co-exist with stent under-expansion or ensue independently. Malapposition can occur in different stages (acute, post-procedural, or late stage), possibly due to the underlying vascular process of inflammation and/or remodeling of the vessel wall. When the same lesions were evaluated by both IVUS and OCT, acute malapposition was detected by OCT more than twice as frequently as with IVUS: 14% versus 39% in OPUS-CLASS (Optical coherence tomography compared to intravascular ultrasound in coronary lesion assessment study) [71].

Although an acute stent malapposition can be easily detected with OCT, as seen in Figure 5, both in transverse and longitudinal OCT views, its clinical outcomes remain uncertain. Several studies have reported that an acute stent malapposition is not associated with adverse clinical outcomes [72].

### 6.3. Stent Edge Dissection

Stent edge dissections, typically detected by OCT, are defined as linear rims of tissue adjacent to a stent edge (<5 mm) with a width of ≥200 μm. Different factors can affect the edge dissection, including the dissection depth/location/length, dissection flap angle, and residual lumen area at the dissection site: meaningful edge dissection, defined as lumen narrowing less than 4 mm^2^ or dissection angle ≥60°, has been associated with an increased incidence of early ST [46].

### 6.4. Longitudinal Stent Deformation

It is defined as shortening or elongation of the stent along its longitudinal axis after deployment. It is under-recognized on angiography, and imaging-based studies have reported an incidence of up to 1%. A post-stent implantation IVUS analysis detected three different patterns of longitudinal stent deformation: deformation with intra-stent wrinkling and overlapping of the proximal and distal stent fragments within a single stent, deformation with elongation, and deformation with shortening. The phenomenon can occur secondary to a variety of mechanisms, and identification is important as, if left untreated, it may be associated with a risk of ST [73].

### 6.5. Tissue Protrusion

Stent implantation may be associated with TP (e.g., plaque or thrombus) into the lumen through device struts. This scenario occurs especially in unstable lesions, but its clinical impact is unknown [74,75]. IVUS could not detect TPs in half of the patients due to its limited resolution compared to OCT. OCT-detected TPs can be classified into three categories according to the extent of the vessel injury: smooth protrusions with minimal vessel injury, disrupted fibrous TPs with mild vessel injury, and irregular protrusions with moderate to severe vessel injury and a high possibility of medial disruption and a lipid core penetration, which has been associated with target vessel failure.

The volume of the protruding tissue as viewed by OCT is associated with an unstable plaque feature and peri-procedural MIs. The CLI-OPCI and Harmonizing Outcomes with Revascularization and Stents in Acute Myocardial Infarction (HORIZONS-AMI) sub-studies showed that TPs during ACS are more likely associated with early ST than those in non-ACS patients in the clinical setting [76].

### 6.6. Slow Flow/No-Reflow

The slow flow/no-reflow phenomenon is defined as the worsening of TIMI (Thrombolysis in Myocardial Infarction) flow on angiography during the PCI procedure in the absence of mechanical obstruction. The event is mainly due to the distal embolization of thrombus and/or lipidic components of the plaque; the subsequent microvascular obstruction results in a reduced epicardial flow without significant epicardial stenosis.

This phenomenon is a strong predictor of 5-year death of patients who undergo PCI for STEMI, and generally it is associated with worse in- and out-of-hospital outcomes [77,78].

In ACS, the slow flow is generally due to the thrombus burden that could also be evaluated with the standard angiography, while in the chronic setting, where the embolism comes from a lipid-rich plaque, imaging (OCT, IVUS, computed tomography-SCAN) is crucial. Identifying the most vulnerable plaque should suggest the use of an embolic protection device. One of the most used devices, the FiltrapTM, has been shown to reduce the incidence of slow-flow phenomenon and in-hospital adverse clinical events [79]. Both IVUS and OCT are valuable tools; thus, the major concern about IVUS is the difficulty in discerning thrombus vs. high lipid-burden plaque due to echo attenuation.

The NIRS technology applied to the IVUS images can distinguish the lipid plaque from thrombus and give us a metric value to quantify the lipid burden.

The lipid core burden index (LCBI) and its derivative parameters are the most used predictors of slow flow. Sato et al. used the total LCBI/highest LCBI ratio within the culprit plaque to predict filter-no reflow in ACS patients [80].

Suzuki et al. used the maximum 4 mm lipid core burden index (maxLCBI4 mm), and the best cut-off to identify the vulnerable plaque resulting in slow flow was maxLCBI4 mm >600 in ACS and maxLCBI4 mm >400 in chronic settings (the sensitivity was 100% and specificity was ≈65%) [81].

The OCT better allows a complete evaluation of the plaque morphology, even though it cannot correctly evaluate the lipid burden in complex atheromatous plaques; the best predictors of slow flow are considered the number of cholesterol crystals (CCs), the lipid arch, and the thin cap fibrous atheroma. CCs are more frequently visible in advanced atheromatous coronary plaque, suggesting a role of CCs as a marker of complex plaque [82].

Furthermore, CCs could be directly responsible for the distal embolization in the no-reflow phenomenon.

An analysis of Katayama et al. selected the combination of the lipid arc > 139° and the number of CCs > 12 for predicting the no-reflow phenomenon in ACS (sensitivity 48%, specificity 93%, and accuracy 86%, respectively) [83] (Table 3).

The table summarizes key morphological and compositional plaque features identifiable by intravascular imaging modalities (OCT, IVUS, and NIRS-IVUS) that are associated with increased risk of no-reflow during or after PCI. Parameters such as lipid arc >180°, lipid length >4 mm, microchannels >12, and maxLCBI4mm >400 are consistently linked with plaque vulnerability, distal embolization, and microvascular obstruction. Legend: ↑ augmented.

### 6.7. Geographic Miss

GM is defined as residual disease or dissection at the stent edge, which is associated with a high rate of restenosis and major adverse events [38,84]. Angiography has well-established limitations for the identification of GM [85,86].

GM could be categorized as longitudinal or radial. The first is defined as an injured segment of the vessel not covered by a stent; the latter is described as a balloon/artery size ratio <0.9 or >1.3 [87]. Two recent studies evaluated the role of ACR-OCT on the incidence of GM.

As outlined above, the OPTICO-integration II trial randomized 84 consecutive patients to ACR-OCT-guided PCI, OCT-guided PCI, and angiography-alone PCI.

Rates of primary endpoint (composite of longitudinal GM and major dissection assessed by post-PCI OCT) were 4.2% in the ACR-OCT-guided PCI, 17.0% in the OCT-guided PCI, and 22.9% in the angiography-alone PCI group. Even though this study demonstrated the superiority of ACR-guided PCI in reducing the incidence of GM, the clinical impact of this evidence was not evaluated [15]. Nevertheless, a similar study by Koyama et al. comparing ACR with OCT-guided PCI did not confirm these findings in terms of longitudinal GM (27.6% with ACR vs. 34.0% with OCT) but established a 50% reduction in the risk of distal stent edge dissection after ACR-guided PCI [88].

So far, no randomized clinical trial has been performed to evaluate the impact of IVUS on the incidence of GM.

## 7. Intravascular Imaging in Coronary Stent Restenosis

Among the 5,000,000 patients of the Diagnostic Catheterization and Percutaneous Coronary Intervention registry of the National Cardiovascular Data Registry (NCDR) between 2009 and 2017, 10.6% of patients underwent PCI for ISR lesions [89].

IVUS could be useful in the process of evaluating ISR: suggested classification distinguishes ISRs as focal (restenosis ≤10 mm in length), multifocal (≥1 focal lesions), and diffuse (luminal narrowing >10 mm in length) with or without stent edge involvement [90]. This classification is superimposable to the angiographic ISR classification provided earlier by Mehran et al., which incorporated four angiographic types: focal, in-stent diffuse, diffuse proliferative, and total occlusion [91].

Goto et al. adopted IVUS to compare the ISR mechanisms of bare metal stents, first-generation DES, and second-generation DES. By IVUS analysis, they found that less neointimal hyperplasia, smaller stent areas, longer stent lengths, and more stent fractures were more common ISR features of first- and second-generation DES than bare metal stent restenosis [92].

Restenosis is a stable, scar-like healing response in which smooth muscle cell proliferation and extracellular matrix growth narrow the stented segment, typically occurring within the first year after PCI. In contrast, neo-atherosclerosis represents the development of true atherosclerotic plaque inside the stent, and it has been identified as one of the pathogenetic characteristics of ISR or late ST; the mechanisms underlying its rapid development and its association with stent failure are currently unknown [93]. Histologically, neo-atherosclerosis is characterized by the accumulation of lipid-laden foamy macrophages within the neointima, with or without necrotic core formation and/or calcification [67]. The development of neo-atherosclerosis may occur in months to years following stent placement. The prevalence and characteristics of in-stent neo-atherosclerosis have been investigated by data acquired from intravascular imaging modalities, including angiography, IVUS, and OCT or optical frequency domain imaging. Optical coherence tomography, or optical frequency domain imaging, has superior axial resolution (10–20 µm), enabling better characterization of neointimal tissue within stents, providing the opportunity to understand the pattern and the mechanisms of ISR development.

Three OCT neointima patterns have been described: homogenous neointima, layered neointima, and heterogeneous neointima. The first one is characterized by a high-backscattering and low-attenuation image due to its content of smooth muscle cells and collagen [94]. Layered neointima, as its name suggests, confers to the neointima a multilayered aspect with a high-backscattering superficial layer; instead, the deeper layers present low-backscattering properties. The speckled pattern of areas of high and low backscattering is the main feature of heterogeneous neointima that may be determined by thrombus presence and active inflammation cells, like macrophages, as histopathological studies have proven [95,96]. Another potential cause of lower signal areas within the neointima on OCT imaging is granulation tissue or organized thrombus consisting of extracellular matrix and angiogenesis with varying degrees of inflammatory cell infiltrate, which is diffusely or focally seen in the deep neointima around stent struts.

Findings from IVUS-based tissue characterization should be interpreted with caution because the technology lacks sufficient resolution (spatial resolution = 150–250 µm) to enable reliable determination of plaque composition. However, various IVUS-guided analyses of neo-intima patterns allowed the collection of more accurate records on both the development history and tissue characterization of ISR. A virtual histology IVUS assessment of restenotic neointimal tissue in bare metal stents *(n=* 47, mean = 43.5 months) and DES *(n* = 70, mean = 11.1 months) revealed that duration of implant correlated positively with percent necrotic core (R = 0.35) and percent dense calcium (R = 0.57), providing further evidence that the prevalence of neo-atherosclerosis increases with time both in bare metal stents and DES [97].

The factors that predispose individuals to neo-atherosclerosis, and their overlap with risk factors for native atherosclerosis, remain poorly defined, and effective treatment strategies to prevent this complication have yet to be established.

Recognition of specific imaging patterns—such as focal under-expansion, heterogeneous tissue with lipid-laden or calcified neo-intima, or spotty malapposition—should prompt additional lesion preparation; suspicion of neo-atherosclerosis arises with OCT/IVUS evidence of in-stent lipid or calcific changes, and these features help guide therapy selection, including balloon sizing, atherectomy, or use of drug-coated devices.

Future studies are warranted to better identify patients at risk of developing neo-atherosclerosis after stent implantation.

## 8. Spontaneous Coronary Artery Dissection and Imaging Evaluation

Spontaneous coronary artery dissection (SCAD) is defined as a non-atherosclerotic, non-traumatic, or iatrogenic separation of the coronary arterial layers that leads to myocardial ischemia due to a coronary compression secondary to intimal tear or due to a vasa vasorum hemorrhage [98]. The clinical presentation is superimposable to an MI, and it accounts for up to 4% of all ACS, being more frequent in specific recipients (e.g., pregnant women or patients with a history of fibromuscular dysplasia) [99,100].

The Yip-Saw angiographic classification describes three types of SCADs: type one is characterized by contrast dye staining of the arterial wall with multiple radiolucent lumens; type two has a long, diffuse, and smooth narrowing angiographic appearance; and type three has an angiographic appearance of a focal or tubular stenosis that mimics atherosclerosis [101,102,103].

For their capacity to examine the arterial wall ultrastructure and its intimal–medial contents, OCT and IVUS guarantee a specific diagnosis of SCAD in contrast to traditional angiography.

OCT represents the best intracoronary imaging modality for SCAD evaluation for both diagnostic and therapeutic purposes. Indeed, IVUS cannot localize intimal tears due to lower spatial resolution, but it can identify vasa vasorum hemorrhage [104]. With its higher resolution, OCT allows for a precise intimal tear identification and distribution of the dissection flap, the magnitude of luminal stenosis, side branch involvement, and the possible presence of thrombus. Although OCT provides the highest spatial resolution and is often considered the most detailed modality for visualizing SCAD anatomy, its use must be carefully balanced against procedural risks: because OCT requires contrast injection to clear blood, it carries a risk of iatrogenic dissection propagation, especially in fragile or long lesions. In such cases, IVUS may be preferred, as it does not require contrast flushing and offers adequate visualization to confirm diagnosis and guide management.

In a prospective study in which 17 patients with angiographically suspicious SCAD were selected from more than 5000 patients undergoing coronary angiography, OCT confirmed the SCAD diagnosis, detecting pathological features of SCAD that were angiographically silent as intimal rupture sites, thrombi, and intramural hematoma [105].

## 9. Future Perspectives

Looking ahead, the integration of advanced technologies is expected to redefine the role of intravascular imaging in both research and clinical practice. Artificial intelligence (AI)–based algorithms for automated segmentation, plaque characterization, and lesion quantification are poised to enhance both the accuracy and efficiency of IVUS and OCT analysis, enabling real-time guidance and standardized interpretation during coronary interventions [106,107]. The advent of hybrid IVUS–OCT platforms represents another major step forward, combining the deep penetration of IVUS with the superior resolution of OCT to provide a comprehensive assessment of vessel architecture, plaque composition, and stent–vessel interactions within a single acquisition [108,109]. From an engineering standpoint, ongoing efforts focus on miniaturization, precise co-registration, and catheter robustness to ensure clinical feasibility. Emerging modalities such as polarization-sensitive OCT (PS-OCT) further refine tissue characterization by identifying collagen-rich and fibrotic regions, which may improve assessment of plaque vulnerability and healing [110,111]. In parallel, photoacoustic imaging (PAI) introduces molecular contrast for mapping lipid and hemoglobin content, offering functional information beyond morphology [112]. Collectively, these innovations are driving a shift toward clever, multimodal intravascular imaging systems that integrate structural, compositional, and functional data—advancing precision-guided coronary intervention and personalized cardiovascular care.

## 10. How to Choose Between IVUS and OCT

**Figure 6:**
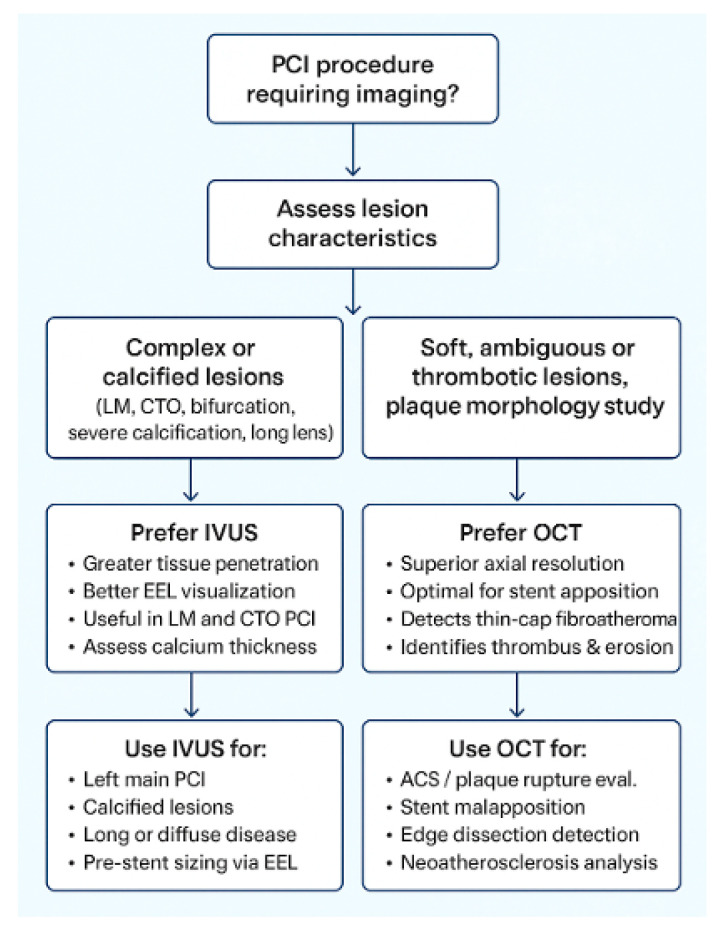
Practical algorithm for selecting intravascular ultrasound (IVUS) or optical coherence tomography (OCT) during percutaneous coronary intervention (PCI) guidance. Abbreviations: LM, left main; CTO, chronic total occlusion; EEL, external elastic lamina; ACS, acute coronary syndrome.

Selection between IVUS and OCT should be guided by clinical context, vessel characteristics, and procedural goals (Figure 6).

OCT is unequaled for high-resolution plaque and lumen detail (thin cap fibroatheroma, cholesterol crystals, tissue prolapse, edge dissection, and stent-strut apposition) and for EEL-based stent sizing when clear lumen images are obtainable, but it requires blood clearance (contrast or flush) and so may be limited by renal dysfunction/contrast-load concerns or by long/fragile dissections where contrast can propagate harm. 

IVUS, in contrast, offers deeper tissue penetration and greater utility in larger or calcified vessels. It is particularly valuable for characterizing calcium depth and arc, planning calcium modification strategies (atherectomy or intravascular lithotripsy), and performing accurate EEL-based sizing in the left main or ostial lesions. IVUS does not require contrast and is therefore preferable in patients with renal dysfunction or when minimizing contrast load is essential. In complex lesion subsets, IVUS facilitates assessment of plaque burden and vessel geometry in bifurcations, CTOs, and diffusely calcified segments, whereas OCT excels in evaluating side-branch ostia, detecting stent malapposition, and preventing geographic miss with angiographic co-registration. From a practical perspective, OCT involves higher contrast use, greater consumable cost, and may extend procedural time, whereas IVUS offers broader availability and simpler logistics. When used appropriately, both modalities yield comparable clinical outcomes; thus, selection should balance diagnostic needs, renal function, vessel characteristics, and operator expertise.

## 11. Conclusions

Both IVUS and OCT represent a remarkable technological advancement in the field of interventional cardiology. A substantial body of evidence has established their role in the individuation of a correct diagnosis when angiography alone could not guarantee reliable guidance, and their role as a PCI optimization tool is well known. 

Nonetheless, the prognostic superiority of one modality over the other in improving long-term clinical outcomes is still debated, with most studies demonstrating comparable reductions in stent thrombosis and target lesion failure when either is used properly.

Furthermore, integration of multimodal imaging (e.g., IVUS–OCT co-registration, NIRS-IVUS) and AI-assisted image analysis is likely to refine lesion assessment and procedural decision-making even further. The future of PCI guidance lies in personalized, data-driven imaging strategies that combine anatomical, functional, and biological insights to achieve optimal revascularization outcomes.

## Figures and Tables

**Table 1:** Physical characteristics and principles of intravascular ultrasound (IVUS) and optical coherence tomography (OCT).

Manufacturer	Source	Axial Resolution (µm)	Lateral Resolution (µm)	Tissue Penetration (mm)	Frame Width (mm)	Pullback Distance (mm)
***IVUS***						
*Boston Scientific* *(OptiCross)*	40 MHz US	38	80–200	>5	0.02–0.03	100
*Volcano/Philips* *(Eagle Eye vs. Refinity)*	20 MHz US	170	100	>5	0.02–0.03	150
	45 MHz US	46	179	>5	0.02–0.03	150
*InfraReDx/Nipro*	50 MHz US	20	240–260	8	0.02–0.07	150
*Boston Scientific* *(OptiCross HD)*	60 MHz US	22	50–150	>5	0.02–0.03	100
*ACIST HDi*	60 MHz US	40	90	3	0.02–0.17	120
*Terumo* *(Altaview)*	60 MHz US	<30	100	>6	0.02–0.1	150
***OCT***						
*Abbott* *(Dragonfly OPTIS)*	Near-infrared	<20	20–40	1 to 2	0.1–0.2	75
*Terumo* *(Lunawave)*	Near-infrared	<20	30	1 to 3	0.13–0.25	150

**Table 2:** Main findings from leading trials comparing intravascular-imaging-guided percutaneous coronary intervention (PCI) versus traditional angiography-guided procedures. Abbreviations: IVUS, intravascular ultrasound; OCT, optical coherence tomography; MACE, major adverse cardiac events; HR, hazard ratio; MI, myocardial infarction; CI, confidence interval; MD, mean difference; ST, stent thrombosis; TVF, target vessel failure; TL-MI, target-lesion myocardial infarction; TLR, target-lesion revascularization; TVR, target vessel revascularization.

Trial	*N*	Intervention	Comparator	Mean Follow-Up	Key Results
**AVIO (2013)**	284	IVUS-guided PCI	Angiography guidance	24 months	Post-procedure, in the lesion minimal lumen diameter showed a statistically significant difference in favor of the IVUS group (2.70 mm ± 0.46 mm vs. 2.51 ± 0.46 mm; *p* = 0.0002)
**ADAPT-DES (2014)**	8583	IVUS-guided PCI	Angiography guidance	1 year	Reduced 1-year rates of definite/probable ST (0.6% vs. 1.0%; HR 0.40, 95% CI 0.21–0.73; *p* = 0.003), MI (2.5% vs. 3.7%; HR 0.66, 95% CI 0.49–0.88; *p* = 0.004), and MACEs (cardiac death, MI or ST) (3.1% vs. 4.7%; HR 0.70, 95% CI 0.55–0.88; *p* = 0.002)
**CLI-OPCI (2015)**	1002	OCT-guided PCI	Angiography guidance	319 days	Suboptimal stent implantation in 31.0% of lesions, with increased incidence of MACE in the angiography guidance group (59.2% vs. 26.9%; *p* < 0.001)
**DOCTORS (2016)**	240	OCT-guided PCI	Angiography guidance	6 months	Significantly higher fractional flow reserve value (0.94 ± 0.04 vs. 0.92 ± 0.05, *p* = 0.005)
**ILUMIEN III (2016)**	450	OCT-guided PCI	Angiography guidance IVUS	1 year	Final median minimum stent area was 5.79 mm^2^ with OCT guidance, 5.89 mm^2^ with IVUS, and 5.49 mm^2^ with only angiography guidance, showing how OCT guidance was non-inferior to IVUS guidance (one-sided 97.5% lower CI −0.70 mm^2^; *p* = 0.001), but not superior *(p* = 0.42)
**ILUMIEN IV (2023)**	2487	OCT-guided PCI	Angiography guidance	2 years	Two primary efficacy endpoints: minimum stent area after PCI of 5.72 ± 2.04 mm^2^ in the OCT cohort and 5.36 ± 1.87 mm^2^ in angiography group (MD 0.36 mm^2^; 95% CI 0.21–0.51; *p* < 0.001); TVF in 88 vs. 99, OCT vs. angiopraphy cohorts (HR, 0.90; 95% CI, 0.67–1.19; *p* = 0.45)
**OCTOBER (2023)**	1201	OCT-guided PCI	Angiography guidance	2 years	MACEs (composite of death from a cardiac cause, TL-MI, ischemia-driven TLR) had occurred in 59 patients (10.1%) over the intervention group vs. 83 patients (14.1%) in angiography guidance (HR 0.7; 95% CI 0.5–0.98; *p* = 0.035)
**OCTIVUS (2023)**	2008	OCT-guided PCI	IVUS-guided PCI	1 year	The primary endpoint (a composite of death from a cardiac cause, TL-MI, ischemia-driven TLR) had occurred in 25 patients (2.5%) over the OCT-group vs. 31 patients (3.1%) in IVUS-guidance (absolute difference, −0.6 percentage points; upper boundary of one-sided 97.5% CI, 0.97 percentage points; *p* < 0.001)
**RENOVATE COMPLEX PCI (2023)**	1639	OCT and IVUS-guided PCI	Angiography guidance	2 years	The primary endpoint (composite of death from cardiac causes, target vessel-related MI, or clinically driven TVR) had occurred in 76 patients (cumulative incidence, 7.7%) in the intravascular imaging group and in 60 patients (cumulative incidence, 12.3%) in the angiography group (HR 0.64, 95% CI 0.45–0.89; *p* = 0.008)
**OCCUPI** **(2024)**	1604	OCT-guided PCI	Angiography guidance	1 year	The primary endpoint (composite of cardiac death, ischemia-driven TLR, MI, stent thrombosis) for OCT-guided vs. angiography-guided PCI was: 5% vs. 7% (HR 0.62, 95% CI 0.41–0.938, *p* = 0.023)

**Table 3:** Predictive intravascular imaging biomarkers of no-reflow.

Predictive Biomarker	Threshold/Definition	Mechanistic Implication	Clinical Relevance (No-Reflow Risk)
Lipid arc	>180°	Large lipid pool prone to distal embolization	↑ Risk of no-reflow post-percutaneous coronary intervention
Lipid length	>4 mm	Extensive lipidic plaque burden	↑ Risk of microvascular obstruction
Thin-cap fibroatheroma (TCFA)	Fibrous cap < 65 μm	High plaque vulnerability	↑ Risk of distal emboli
Macrophage accumulation	Qualitative high signal with shadowing	Active inflammation	↑ Risk of slow-/no-reflow
Plaque rupture or cavity	Visible cavity or disrupted cap	Embolization source	↑ No-reflow likelihood
Attenuated plaque	Deep ultrasound attenuation without calcification	Necrotic core, lipid-rich	↑ Risk of no-reflow
Lipid Core Burden Index (maxLCBI4mm)	>400	High lipid content segment	↑ No-reflow and periprocedural myocardial infarction
Microchannels (CCs)	>12 channels	Neovascularity, plaque instability	↑ Risk of no-reflow
Thrombus burden	Large filling defect or thrombus length >5 mm	Embolic potential	↑ Risk of no-reflow

## Abbreviations

The following abbreviations are used in this manuscript:

ACR	angiographic co-registration
ACS	acute coronary syndrome
AI	artificial intelligence
CAD	coronary artery disease
CCs	cholesterol crystals
CI	confidence interval
CTO	chronic total occlusion
DES	drug-eluting stent
EEL	external elastic lamina
FCT	fibrous cap thickness
FFR	fractional flow reserve
GM	geographic miss
ISR	in-stent restenosis
IVUS	intravascular ultrasound
LCBI	lipid core burden index
LM	left main
MI	myocardial infarction
MLA	minimum lumen area
MLD	minimum lumen diameter
MSA	minimum-stent area
NIRS	near-infrared spectroscopy
OCT	optical coherence tomography
OMT	optimal medical therapy
PCI	percutaneous coronary interventions
SCAD	spontaneous coronary artery dissection
ST	stent thrombosis
TCFA	thin cap fibroatheroma
TIMI	Thrombolysis in Myocardial Infarction
TP	tissue protrusion
TV-MI	target vessel-myocardial infarction
TVR	target vessel revascularization
